# Electrostatic force spectroscopy revealing the degree of reduction of individual graphene oxide sheets

**DOI:** 10.3762/bjnano.9.106

**Published:** 2018-04-11

**Authors:** Yue Shen, Ying Wang, Yuan Zhou, Chunxi Hai, Jun Hu, Yi Zhang

**Affiliations:** 1 Key Laboratory of Comprehensive and Highly Efficient Utilization of Salt Lake Resources, Qinghai Institute of Salt Lakes, Chinese Academy of Sciences, Xining, Qinghai 810008, China; 2 Key Laboratory of Salt Lake Resources Chemistry of Qinghai Province, Xining 810008, China; 3 Key Laboratory of Interfacial Physics and Technology, Shanghai Institute of Applied Physics, Chinese Academy of Sciences, Shanghai 201800, China

**Keywords:** degree of reduction, dielectric property, electrostatic force microscopy, electrostatic force spectroscopy, graphene oxide

## Abstract

Electrostatic force spectroscopy (EFS) is a method for monitoring the electrostatic force microscopy (EFM) phase with high resolution as a function of the electrical direct current bias applied either to the probe or sample. Based on the dielectric constant difference of graphene oxide (GO) sheets (reduced using various methods), EFS can be used to characterize the degree of reduction of uniformly reduced one-atom-thick GO sheets at the nanoscale. In this paper, using thermally or chemically reduced individual GO sheets on mica substrates as examples, we characterize their degree of reduction at the nanoscale using EFS. For the reduced graphene oxide (rGO) sheets with a given degree of reduction (sample n), the EFS curve is very close to a parabola within a restricted area. We found that the change in parabola opening direction (or sign the parabola opening value) indicates the onset of reduction on GO sheets. Moreover, the parabola opening value, the peak bias value (tip bias leads to the peak or valley EFM phases) and the EFM phase contrast at a certain tip bias less than the peak value can all indicate the degree of reduction of rGO samples, which is positively correlated with the dielectric constant. In addition, we gave the ranking of degree for reduction on thermally or chemically reduced GO sheets and evaluated the effects of the reducing conditions. The identification of the degree of reduction of GO sheets using EFS is important for reduction strategy optimization and mass application of GO, which is highly desired owing to its mechanical, thermal, optical and electronic applications. Furthermore, as a general and quantitative technique for evaluating the small differences in the dielectric properties of nanomaterials, the EFS technique will extend and facilitate its nanoscale electronic devices applications in the future.

## Introduction

Graphene is a two dimensional (2D) crystal with superior mechanical [1], thermal [2], electrical [3–4] and optical [5] properties. It can be produced using graphene oxide (GO) as a precursor through cost-effective reduction methods with high yield. Reducing GO to reduced graphene oxide (rGO) is a key step toward the large-scale use of graphene [6]. Different reduction processes that partially restore the structure and properties result in different properties of rGO, which in turn affect the final performance of rGO-based devices [6].

Because the microstructure and properties of GO sheets can be greatly manipulated during the reduction process, it is important to characterize and evaluate the reducing effect of different reduction processes. Microelectrode-based electrical conductivity measurements [7] and spectral methods, such as X-ray photoelectron spectroscopy (XPS) [8–9], Raman spectroscopy [10], and UV–vis absorption spectra [11–12], reflect the average information of rGO materials. However, they cannot characterize an isolated rGO sheet at the nanoscale. Optical observation [13] and transmission electron microscopy (TEM) [14] has shown color changes and atomic scale feature changes, respectively, in GO sheets after reduction. However, any changes in performance are not identified, and differentiating rGO sheets with a similar degree of reduction is difficult. Based on the changes in the electrical properties of rGO, scanning probe microscopy (SPM) has also been employed recently to study the reduction of GO sheets at the nanoscale. Conductive atomic force microscopy (CAFM) [15–16] can be used to verify the reduced nanostructures on GO sheets. However, because CAFM relies on contact with the sample, the electrically induced reduction or oxidation may affect the degree of reduction of rGO sheets [17]. Scanning polarization force microscopy (SPFM), also called dielectric force microscopy, was developed firstly by Hu et al. in 1995 [18–19] to measure the dielectric properties of soft or weakly bound materials on a substrate that could be easily damaged by a scanning tip. In the SPFM operation, a direct current (DC) or alternating current (AC) bias is applied to a conductive probe, polarizing the sample below and generating a long-range electrostatic attractive force. This electrostatic attractive force superposes on the van der Waals force between the tip and sample so that both forces contribute to the imaging. In recent years, its applications have been extended to study the local dielectric properties of semiconductor nanomaterials such as GO sheets or partially reduced rGO sheets [20–22], graphene sheets [23], carbon nanotubes (CNTs) [24] and so on. SPFM [25] and electrostatic force microscopy (EFM) [26] have revealed a step-by-step reduction process in GO sheets. However, when the reduction reactions are completed, it is hard for these methods to identify the small difference between GO sheets reduced with different methods.

Although the reduction process of GO sheets has been studied with EFM, little attention has been paid to the use of electrostatic force spectroscopy (EFS) to reveal uniformly reduced GO sheets with various degree of reduction. Previously, EFS was proposed to distinguish graphene domains and optimize EFM imaging [27]. The EFS is a method monitoring the EFM phases with high resolution as a function of the electrical DC bias applied either to the probe or sample. Based on the dielectric constant difference of rGO sheets reduced using various methods, EFS can characterize the degree of reduction of uniformly reduced GO sheets at the nanoscale. Thus, the EFS, which combines imaging and spectroscopy, can be used to supplement SPFM and EFM to further reveal the reducing effects of rGO after the reduction reactions have completed. In this paper, using thermally or chemically reduced GO sheets for examples, we aim to evaluate the degree of reduction of individual rGO sheets at the nanoscale using EFS.

## Results and Discussion

**Figure 1:**
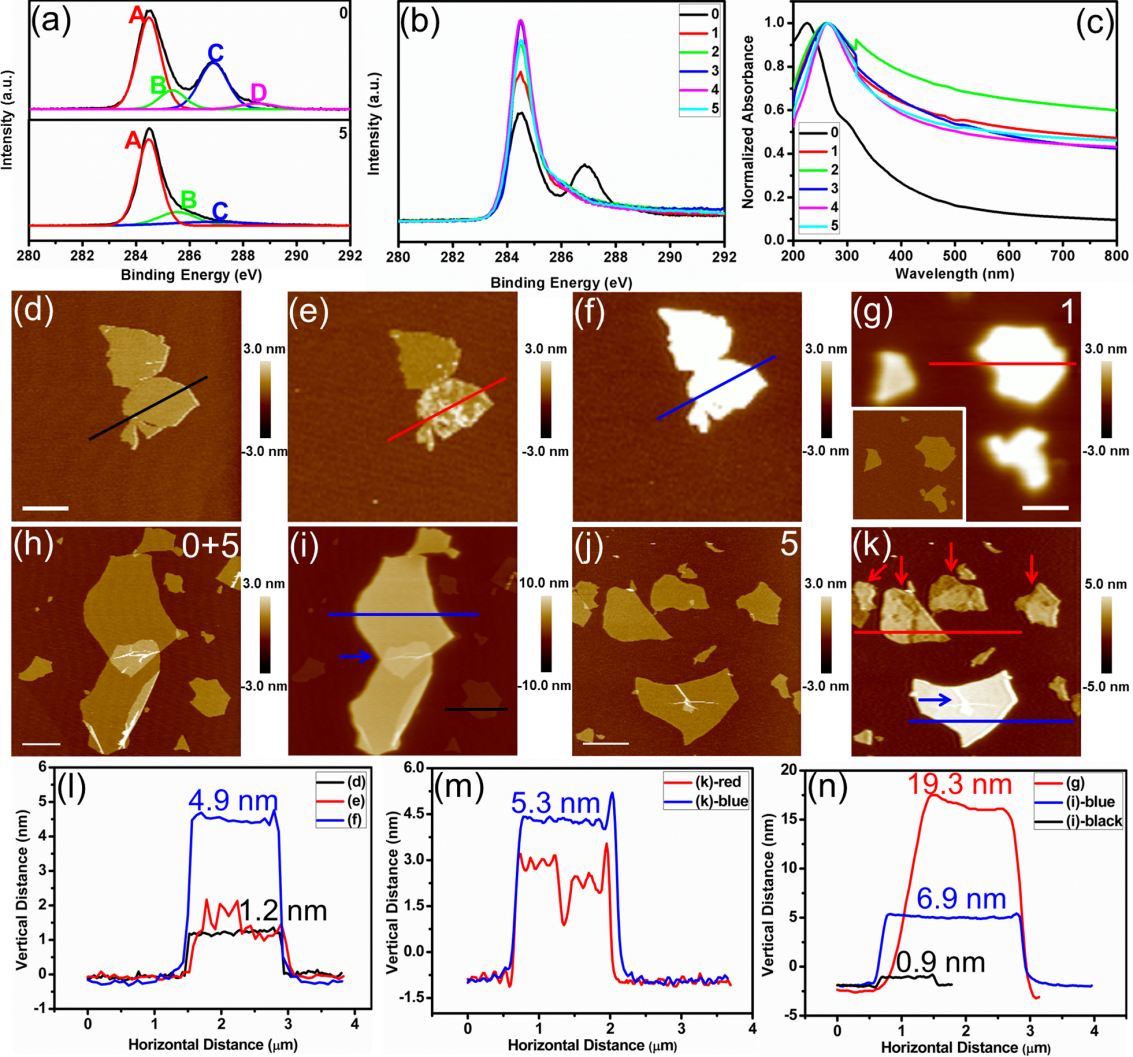
Characterizing the degree of reduction of GO sheets reduced using various methods. C 1s XPS spectra of (a, top) sample 0, (a, bottom) sample 5, and (b) samples 0–5; (c) normalized UV–vis absorption spectra of samples 0–5; in situ SPFM images of GO sheets thermally reduced at 150 °C for (d) 0 min, (e) 15 min, and (f) 75 min; (g) SPFM image of sample 1 (the in situ tapping AFM image is highlighted by the white rectangle); (h) tapping AFM and (i) in situ SPFM images of the mixture of sample 0 and 5; (j) tapping AFM and (k) in situ SPFM images of sample 5; cross-sectional profiles along (l) the lines in (d–f), (m) the lines in (k) and (n) the lines in (g, i). The numbers in the top right corners of the images (g, h, j) represent the sample numbers. The white scale bars represent 1000 nm. The *z*-scale bar is shown to the right of each SPM image.

The thermal or chemical reduction of GO sheets was verified with XPS, UV–vis absorption spectra, and SPFM, as shown in Figure 1. The sample labels and the corresponding descriptions are shown in Table 1. The deconvoluted peaks A–D in Figure 1a centered at the binding energies of 284.5, 285.5, 286.9, and 288.5 eV, respectively, correspond to C=C/C–C in aromatic rings, C–O (epoxy and alkoxy), C=O, and COOH groups, respectively [20]. After reduction (sample 5), the XPS peak magnitudes for carbon atoms bonded to oxygen have decreased, indicating that most of the oxygen groups have been removed [8–9,20]. From the XPS data, we observed an increase in the ratio of the carbon atoms in aromatic rings (C=C/C–C) to those bonded to oxygen after the chemical reduction [20] from 1.1:1 to 6.3:1 (Figure 1a). However, the degree of reduction of samples 1–5 cannot be characterized from the almost coincident XPS spectrum in Figure 1b. In the normalized UV–vis absorption spectra (Figure 1c), the red-shift of the main absorbance peak from 226 nm to 264 nm the absorption increment in the visible region as well as the disappearance of the broad shoulder at 300 nm for samples 1–5 compared to sample 0 imply the removal of the oxygen-containing groups and the restoration of the π-conjugation network within the graphene nanosheets [11–12]. Nevertheless, except for the transparency injury in sample 2 caused by high temperature (450 °C) and oxidation in the atmosphere, no other differences amongst samples 1–5 were observed.

**Table 1:** Sample labels and corresponding descriptions.

Sample label “n”	Sample description

0	GO sheets
1	thermally reduced GO sheets at 200 °C for 15 min
2	thermally reduced GO sheets at 450 °C for 15 min
3	chemically reduced GO sheets with hydrazine monohydrate at 80 °C for 1 h and then thermally reduced at 450 °C for 15 min
4	chemically reduced GO sheets with hydrazine monohydrate at 80 °C for 1 h and then thermally reduced at 200 °C for 15 min
5	chemically reduced GO sheets with hydrazine monohydrate at 80 °C for 1 h

In addition, the increased apparent height in the SPFM images compared with the height in the topography images (Figure 1d–n) indicates that the GO sheets are reduced [21]. We can know whether the reduction is homogeneous (Figure 1f,g,i, blue line shown in Figure 1l, red and blue lines shown in Figure 1n) or heterogeneous (Figure 1e, red arrows marked in Figure 1k, and red lines shown in Figure 1l,m). Thus the initial stage of the reduction process (the reduction occurred on some domains on the GO sheets) can be characterized with SPFM [25]. However, when the GO sheets are reduced uniformly, evaluating the degree of reduction of samples 1–5 from the apparent height is difficult. The SPFM images contain both the dielectric properties and the morphology information (blue arrows marked in Figure 1i,k), indicating the contributions from the polarization force (dielectric properties) and the van der Waals force (topography) between the tip and sample. As we can see in the Figure 1l,m,n, the apparent height of samples 1 and 5 are 19.3 nm (Figure 1n) and 6.9 nm (Figure 1n) or 5.3 nm (Figure 1m), respectively. This inconsistency comes from the apparent height in SPFM imaging being influenced by the changed imaging force (amplitude set point). Considering the deficiencies in the above methods, we further explored EFS to characterize the homogeneously reduced GO samples 1–5 at the nanoscale.

**Figure 2:**
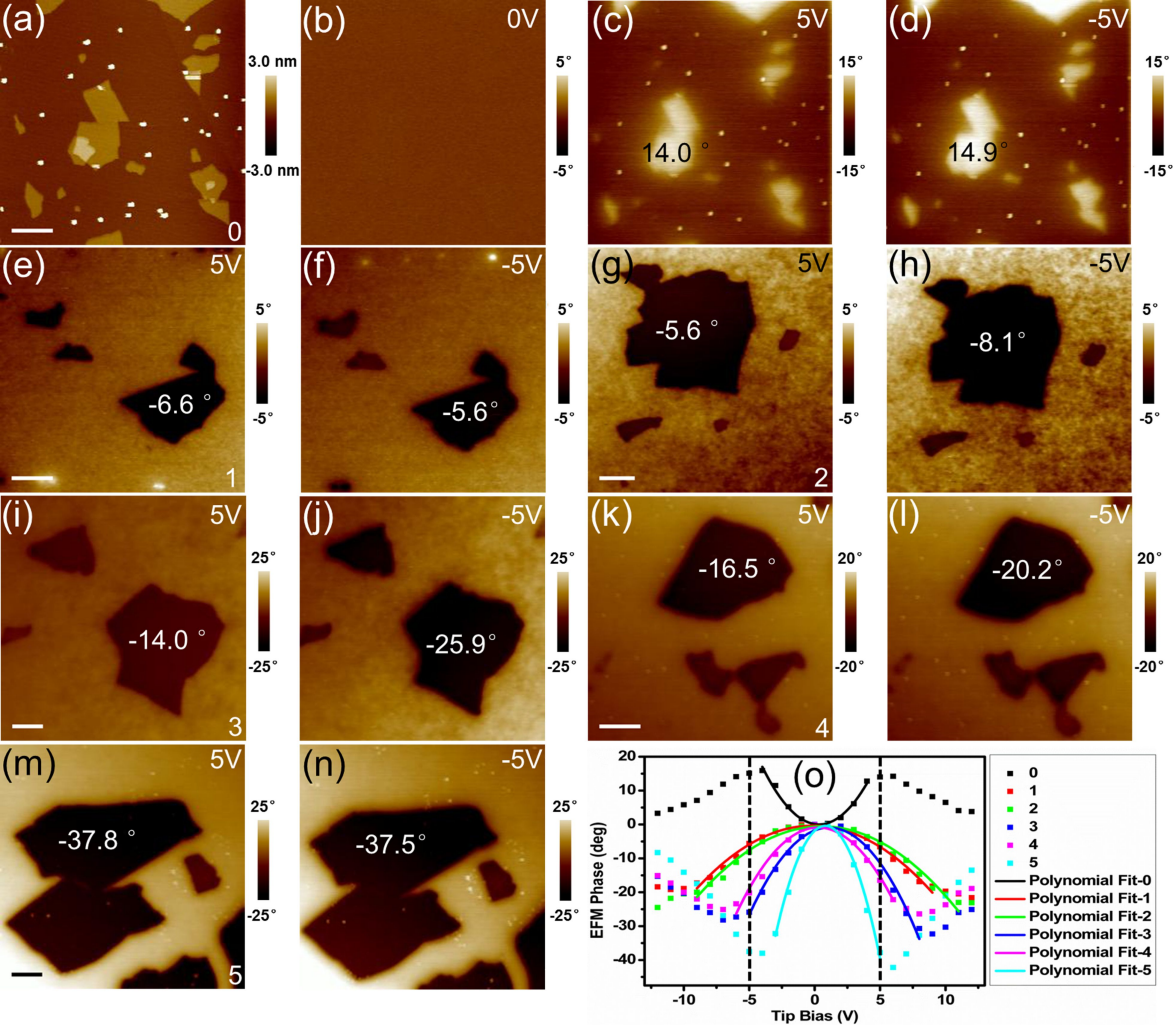
Characterizing the degree of reduction of GO sheets using EFM imaging and EFS: (a) tapping AFM image and in situ EFM images with tip biases of (b) 0 V, (c) 5 V and (d) −5 V of sample 0; EFM images of (e, f) sample 1, (g, h) sample 2, (i, j) sample 3, (k, l) sample 4, and (m, n) sample 5; (o) EFM spectra and the corresponding polynomial fits of samples 0–5 (the dashed vertical lines show the position of the biases at which images (c–n) were recorded). The number in the bottom right corner of the images represents the sample number. The upper right corner shows the tip bias used in the EFM imaging. The EFM phase contrast of the samples is labelled on the corresponding positions. The scale bars represent 1000 nm. The *z*-scale bar is shown to the right of each image.

In order to further characterize the degree of reduction of samples 1–5 at the nanoscale, we tested the EFS of each sample, as shown in Figure 2. EFS is based on measurements of the EFM phase contrast of the sample compared to the mica substrate as a function of different tip biases ranging from −12 V to 12 V. Then, the EFS can be obtained by plotting the EFM phase contrast versus the tip bias.

**Figure 3:**
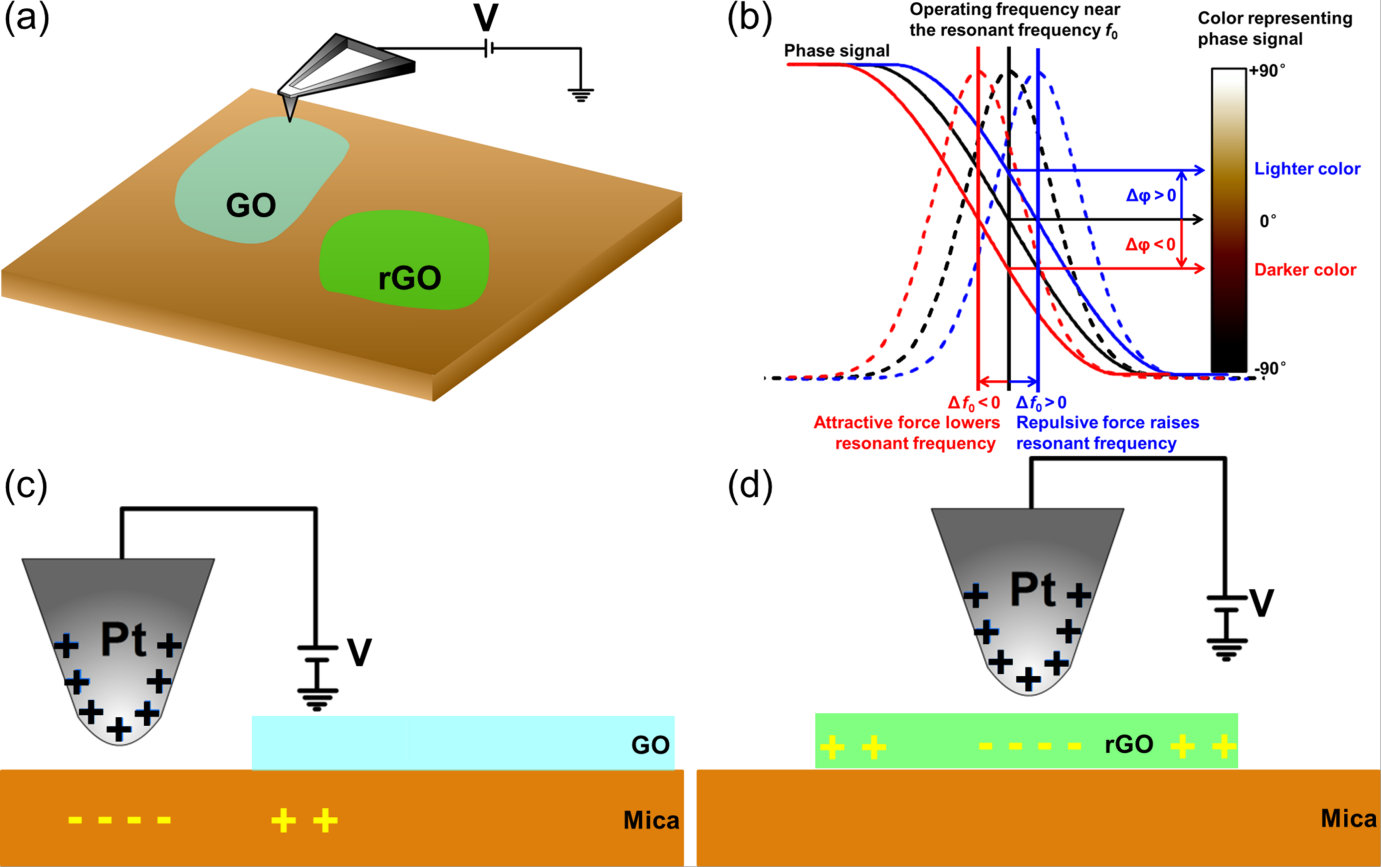
Illustrative diagram of EFM imaging and EFS: (a) schematic depiction of EFM, (b) electrostatic force between the biased tip and sample, causing a phase shift of the cantilever; EFM imaging of (c) GO or (d) rGO on mica substrate.

The principle of using EFS to evaluate the degree of reduction of rGO samples is shown in Figure 3. In a typical EFM measurement, a DC bias voltage is applied to the conductive cantilever (Figure 3a). Because of polarization, opposing charges are induced at the vicinity of the sample surface, causing an attractive force between the tip and the sample, which leads to a phase shift of the cantilever. In the absence of electrical forces, the cantilever has a resonant frequency, *f*_0_. However, the tip bias causes an attractive (or repulsive) electrostatic force, making the cantilever effectively “softer” (“stiffer”), reducing (increasing) the resonant frequency [28–29]. The phase curve then correctly reflects the phase lag between the drive and the cantilever response (Figure 3b) [29]. This correspondingly results in a negative (or positive) phase shift of the cantilever, as labelled with red (or blue) in the Figure 3b. The case of repulsive electrostatic forces (in the parentheses) usually occurs when the sample itself is charged [21]. However, in the experiments here, electrostatic forces between the biased tip and the induced charges on sample surface are only attractive. Thus, in EFM imaging (Figure 3a,c–d), the electrostatic attraction causes a phase shift of the cantilever, leading to a dark color in the contrast (marked with red in Figure 3b).

In the EFS experiments, the probe is biased. The sample, however, which is adhered to the sample holder with insulating double-sided adhesive, is not connected to ground. Thus the tip material has a capacitance similar to an isolated conductor and the rGO sheets or mica act as a dielectric in its electrostatic field, rather than having a capacitance related to the tip–substrate. The tip can be approximated as a spherical conductor with a radius of *R*_Tip_. The tip capacitance *C*_Tip_ can be expressed as:

[1]
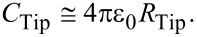


The tip charge, *Q*_Tip_, and the induced charge at the vicinity of the sample surface, *Q*_Sam_, can be expressed respectively as:

[2]
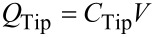


[3]
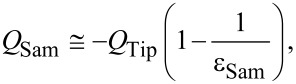


where *V* is the DC voltage applied to the probe, and ε_Sam_ is the dielectric constant of the sample. The forces between the probe and the sample can be expressed as a Coulomb contribution:

[4]
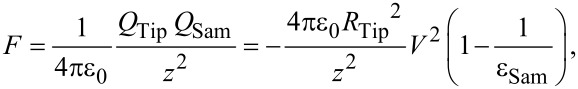


where *z* is the tip–sample distance. By differentiating Equation 4 we obtain the electric force gradients:

[5]
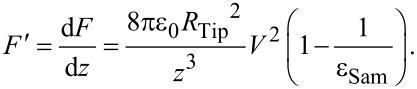


The electric force gradients cause shifts of Δ*f*_0_ in the resonance frequency with a proportional relationship [30]:

[6]
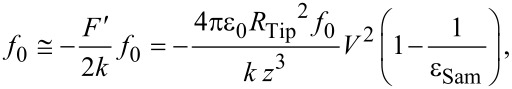


where *k* is the stiffness (or spring constant) of the cantilever. Resonance shifts also give rise to phase shifts, ∆φ, used to generate an image of the electric force gradients. In EFM imaging, the frequency shifts are detected through phase detection, which measures the cantilever’s phase of oscillation relative to the piezo drive [29]. In a small resonant frequency shift range (at low biases), small phase shifts, ∆φ, are proportional to the resonance frequency shifts, Δ*f*_0_:

[7]
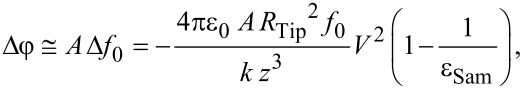


where *A* is a coefficient of proportionality. The contrast between the phase shifts of probe imaging on mica and sample n can be expressed as:

[8]
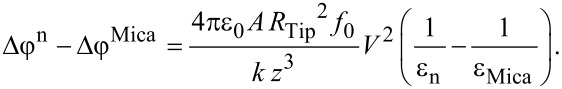


In EFM imaging, the lift mode is used. Topography data recorded during the main pass is used to keep the tip at a constant distance from the surface (lift scan height was 15 nm here) during the interleave trace and retrace, allowing imaging of the long-range electric interactions while minimizing the influence of topography [29]. So the tip–surface distance *z* is a constant, *R*_Tip_, and *f*_0_ and *k* are also kept constant provided that the same cantilever is used. Thus the differences between the phase of the probe on mica and sample n are only related to the tip bias and the local dielectric constant of sample n. For the case of *V* = 0 V for sample 0 (Figure 2b), there is no contrast in the EFM images, consistent with Equation 8 and indicating topographical features virtually absent from the EFM image by using lift mode.

For an rGO sample, when the tip bias increases from 0 V, the EFM contrast is enhanced by the increasing electrostatic attraction gradients. As represented in Figure 2o, within a restricted area, the curve of EFM phase contrast (*Y*_Phase_ = ∆φ^n^ − ∆φ^Mica^) versus tip bias for each sample is very close to a parabola, and fits to the data are expressed respectively as:

[9]
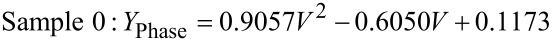


[10]
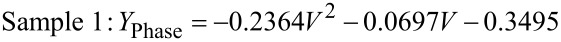


[11]
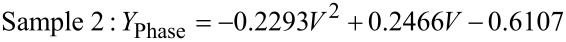


[12]
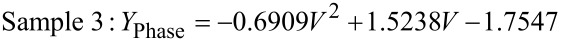


[13]
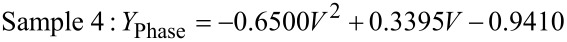


[14]
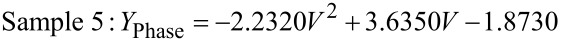


These results provide the parabola opening values (listed later in the article in the Table 2). The parabola opening direction of sample 0 and sample 1–5 is upward (positive parabola opening value) and downward (negative parabola opening values), respectively. The EFM phase of sample 0 is positive (Figure 2c,d,o), whereas samples 1–5 have negative values (Figure 2e–o). We can thus draw the conclusion that ε_GO_ < ε_Mica_ and ε_rGO_ > ε_Mica_ from Equation 8. When ε_Mica_ > ε_GO_, then (Δφ^n^ − Δφ^Mica^) > 0, and the more attractive force gradients between the tip and mica cause the mica substrate to appear darker than the GO sample (Figure 3c). Thus, the GO sample (sample 0) in the EFM images has a lighter color than the mica substrate (Figure 2c,d,o). When ε_Mica_ < ε_rGO_, then (Δφ^n^ − Δφ^Mica^) < 0, and the greater attractive force between the tip and the rGO sample causes the rGO sample to appear darker than the mica substrate (Figure 3d). Thus, the rGO samples (samples 1–5) in the EFM images have a darker color than the mica substrate (Figure 2e–o). Therefore, the sign change (the parabola opening direction change, or color change from lighter to darker) indicates the occurrence of reduction on the GO sheets.

The parabola opening values are negatively correlated with the dielectric constant of the sample on a mica substrate (Equation 8). Thus, according to the parabola opening values, we can deduce the ranking of dergree of the samples' reduction as: sample 0 < sample 1 ≈ sample 2 < sample 4 < sample 3 < sample 5. The parabola of ∆φ^n^ − ∆φ^Mica^ versus tip bias (Equation 8) is consistent with the previous results [31] that the small phase shifts ∆φ can be approximated by

[15]
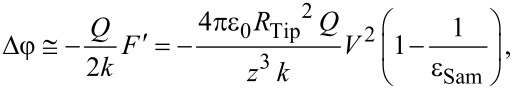


where *Q* is the quality factor of the cantilever. We can obtain the difference between phase shifts of probe imaging on mica and sample n as:

[16]
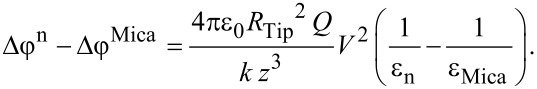


Comparing Equation 16 with Equation 8, the difference is just *A*·*f*_0_ is replaced with *Q.* Therefore, the two expressions are consistent considering both the parameters related to the cantilever. However, the above equation is only valid for the small phase shift angles due to the nonlinearity of the phase shifts with respect to the resonance frequency shifts [31]. The spectroscopic curves deviate strongly from an ideal parabolic shape at high bias Figure 2o.

For a tip bias lower than the peak tip bias values (tip biases lead to the peak or valley EFM phases), the EFM phase contrast is significantly enhanced by the reduction methods used on samples 1–5 (Figure 2e–n, and dashed vertical lines in Figure 2o). This also indicates that the dielectric constant increases from sample 0 to sample 5, improving the electrostatic attraction gradients at certain tip bias. When the tip bias is sufficiently large, the influence of the substrate under the sample will be coupled into the measurement such that the contrast between the sample and mica (after coupling) decreases. For rGO sheets with a strong degree of reduction (high dielectric constants), a small tip bias is sufficient to induce enough local charge in the rGO sheets, polarizing the substrate below, further reducing the electrostatic attraction between the tip and rGO sheets. Thus, a sample with a stronger degree of reduction requires a smaller tip bias to reach the peak EFM phase. The peak tip bias values of each sample (Table 2) gradually decrease from sample 1 to sample 5, indicating a gradually enhanced degree of reduction. Therefore, both the peak tip bias value and the EFM phase contrast at a certain tip bias less than the peak value can indicate the degree of reduction of rGO sheets, which is positively correlated with the dielectric constant. A larger phase contrast and a smaller peak tip bias together indicate a higher degree of reduction. From this logic, we can deduce the ranking of degree of the samples' reduction as: sample 0 < sample 1 ≈ sample 2 < sample 3 ≈ sample 4 < sample 5, which is almost consistent with the quantitative results shown by the parabola opening values. Therefore, chemical reduction with hydrazine monohydrate can reach a higher degree of reduction than thermal reduction without inert atmosphere protection. Moreover, it is not necessary to continue raising the reducing temperature when the GO sheets can be thermally reduced uniformly. For the chemically reduced GO sample (sample 5), further heat treatment in air would oxidize it. Ultimately, to supplement SPFM for characterization of the initial stage of the reduction process, EFS can be used to further identify the degree of reduction of uniformly reduced GO sheets, advancing our understanding of the effects of various reduction methods.

**Table 2:** Peak EFM phase, the corresponding tip biases used in EFM imaging, and parabola opening values (from the EFS measurements in Figure 2o).

Sample	Positive tip bias (V)	EFM phase (°)	Negative tip bias (V)	EFM phase (°)	Parabola opening

0	6	14.2	−4	15.9	0.9057
1	12	−21.6	−11	−19.2	−0.2364
2	12	−23.1	−12	−24.5	−0.2293
3	9	−32.3	−7	−28.2	−0.6909
4	8	−26.4	−7	−25.1	−0.6500
5	6	−42.2	−4	−38.0	−2.2320

## Conclusion

We used EFS to evaluate the degree of reduction of GO sheets that were uniformly reduced by thermal or chemical methods, or through a combination thereof. For the rGO sheets with given degree of reduction (sample n), the EFS curve is very close to a parabola within a restricted area. The sign change of the phase contrast (the parabola opening direction change, or sign change of parabola opening value) indicates the occurrence of reduction on GO sheets. Furthermore, the parabola opening values, the peak tip bias value and the EFM phase contrasts at a certain tip bias less than the peak value can all indicate the degree of reduction of rGO samples, which is positively correlated with the dielectric constant. A smaller parabola opening value, a larger EFM phase contrast and a smaller peak tip bias together indicate a higher degree of reduction. From this logic, we could deduce the ranking of degree of the samples' reduction as: sample 0 < sample 1 ≈ sample 2 < sample 4 < sample 3 < sample 5. From the EFS measurements, we found that chemical reduction with hydrazine monohydrate can enable a higher degree of reduction than thermal reduction without inert atmosphere protection. Additionally, it was found that a further increase in the reduction temperature was not necessary when the GO sheets can be thermally reduced uniformly. For the chemically reduced GO sample, further heat treatment in atmosphere resulted in oxidization. To supplement SPFM for the characterization of heterogeneously reduced GO sheets in the initial stage of the reduction process, EFS can be employed to further identify the degree of reduction of the individual uniformly reduced GO sheets and aids in advancing our understanding of the effects of various reduction methods on GO sheets. The characterization of the degree of reduction of GO sheets using EFS is important for the reduction strategy optimization and mass application of GO material, whose use is strongly desired for its mechanical, thermal, optical and electronic applications. Moreover, we believe that this advanced SPM method provides a general and quantitative approach for characterizing the small differences in the dielectric properties of nanomaterials, which is critically important for further device applications.

## Experimental

The samples under study were GO sheets prepared from graphite powder following a modified Hummers’ method [9,32–35]. Thermal reduction of GO sheets deposited on a substrate was carried out in an oven at 200 °C or 450 °C for 15 min. Chemical reduction of GO was achieved by exposure to a saturated vapor of hydrazine monohydrate in a sealed petri dish at 80 °C for 1 h [36]. Mica substrates were used in this work. The reduction of the GO sheets was verified with XPS (AXIS ULTRA^DLD^, Kratos Analytical, Ltd., Manchester, UK), UV–vis absorption spectra (Lambda 750 UV/VIS/NIR spectrometer, PerkinElmer, Inc., Waltham, MA, USA) and SPFM.

In the SPFM, a DC or AC bias is applied to a tapping mode AFM tip, generating an electrostatic attractive force (polarization force) between the biased tip and the polarized charge on the sample surface. The electrostatic attractive force superposes on the van der Waals force between the tip and sample so that the SPFM imaging gives a higher apparent height than the topographic height of nanomaterials when the dielectric constant of the nanomaterials is larger than the substrate. The apparent height (polarization height) of nanomaterials in SPFM images are an indication of the local dielectric constant difference between the sample and substrate [20,37]. However, when a DC bias is applied, a local net charge (or the surface charge for nanomaterials) would affect the apparent height in SPFM imaging [21–22]. In order to reflect the local variation of the dielectric constant more accurately, we used an AC tip bias (10 V for 100 kHz) instead of a DC one for the SPFM operations in the experiments here.

Tapping AFM, SPFM and EFM were all performed using a commercial AFM instrument (Multimode Nanoscope V, Bruker, previously Veeco) which was installed in an in-house environment-controlled box. The in-house environment-controlled box used here was jointly developed by Shanghai Espec Environmental Equipment Corp. with us. The system uses a compressor and a heater to control the ambient temperature, and uses a compressor and a humidifier to control the humidity of the environment. In order to eliminate the influence of noise from the system, the temperature and humidity generator are physically separated from the AFM. The humidity generator and the AFM are connected with adiabatic hoses, forming the gas transmission loop. The temperature fluctuations were below 0.2 °C and the error of humidity control was about 2% relative humidity (RH). To avoid influences on EFM or SPFM imaging from the dielectric constant change of the mica substrate, all the SPM-based operations were carried out under room temperature at 23 °C and at a relative humidity of 15% in order to ensure constant substrate surface properties (especially regarding the dielectric constant of the mica surface) [20]. In all the SPM operations, the NSC18/Ti-Pt (MikroMasch Co.) tip was used, which employed a silicon cantilever coating with a 10 nm Pt layer on a 20 nm Ti sublayer with a nominal spring constant of ≈3.5 Nm^−1^ and oscillating frequencies of 60–90 kHz. The lift start height and lift scan height in EFM imaging were 20 nm and 15 nm, respectively.
